# Supplementary material for: Memantine in the Prevention of Radiation-Induced Brain Damage: A Narrative Review
Source: Cancers (Basel). 2022 May 31;14(11):2736. doi: 10.3390/cancers14112736 (PMC9179311; doi:10.3390/cancers14112736)
Supplement: Supplementary file 1 [file cancers-14-02736-s001.zip › cancers-1734752-supplementary.pdf]

cancers

MDPI

Table S1. Narrative review checklist.

Section/Topic	#	Checklist Item	Reported on Page
**TITLE**			
Title	1	Identify the report as a Narrative Review	1
**ABSTRACT**			
Unstructured summary	2	Provide an unstructured summary, including, as applicable: background. Objective, brief summary of narrative review and implications for future research, and clinical practice or policy development.	1
**INTRODUCTION**			
Rationale/background	3	Describe the rationale for the review in the context of what is already known.	2
Objectives	4	Specify the key question(s) for the review topic.	2
**METHODS**			
Research selection	5	Specify the process for identifying the literature search (e.g., years considered, language, publication status, study design, and databases of coverage).	2
**DISCUSSION/SUMMARY**			
Narrative	6	Discuss: (1) research reviewed, including fundamental or key findings, (2) limitations and/or quality of research reviewed, and (3) need for future research.	11–13
Summary	7	Provide and overall interpretation of the narrative review in the context of clinical practice for health professionals, policy development and implementation, or future research.	13
